# Photoprotective Bioactivity Present in a Unique Marine Bacteria Collection from Portuguese Deep Sea Hydrothermal Vents

**DOI:** 10.3390/md11051506

**Published:** 2013-05-10

**Authors:** Ana Martins, Tania Tenreiro, Gonçalo Andrade, Mário Gadanho, Sandra Chaves, Marta Abrantes, Patrícia Calado, Rogério Tenreiro, Helena Vieira

**Affiliations:** 1 BIOALVO, SA TEC LABS—Innovation Center, Campus of FCUL, Campo Grande, 1749-016 Lisbon, Portugal; E-Mails: ana.martins@bioalvo.com (A.M.); goncalo.andrade@bioalvo.com (G.A.); marta.abrantes@bioalvo.com (M.A.); patricia.calado@bioalvo.com (P.C.); 2 University of Lisbon, Faculty of Sciences, Centre for Biodiversity, Functional and Integrative Genomics (BioFIG), Campus of FCUL, Campo Grande, 1749-016 Lisbon, Portugal; E-Mails: taniatenreiro@biosurfit.com (T.T.); mariogadanho@biopremier.com (M.G.); sichaves@fc.ul.pt (S.C.); rptenreiro@fc.ul.pt (R.T.)

**Keywords:** deep sea, marine bacteria, natural products, MAR hydrothermal vents, photoprotection

## Abstract

Interesting biological activities have been found for numerous marine compounds. In fact, screening of phylogenetically diverse marine microorganisms from extreme environments revealed to be a rational approach for the discovery of novel molecules with relevant bioactivities for industries such as pharmaceutical and cosmeceutical. Nevertheless, marine sources deliverables are still far from the expectations and new extreme sources of microbes should be explored. In this work, a marine prokaryotic collection from four Mid-Atlantic Ridge (MAR) deep sea hydrothermal vents near the Azores Islands, Portugal, was created, characterized and tested for its photoprotective capacity. Within 246 isolates, a polyphasic approach, using chemotaxonomic and molecular typing methods, identified 23-related clusters of phenetically similar isolates with high indexes of diversity. Interestingly, 16S rRNA gene sequencing suggested the presence of 43% new prokaryotic species. A sub-set of 139 isolates of the prokaryotic collection was selected for biotechnological exploitation with 484 bacterial extracts prepared in a sustainable upscalling manner. 22% of the extracts showed an industrially relevant photoprotective activity, with two extracts, belonging to new strains of the species *Shewanella algae* and *Vibrio fluvialis*, uniquely showing UV-A, UV-B and UV-C protective capacity. This clearly demonstrates the high potential of the bacteria MAR vents collection in natural product synthesis with market applications.

## 1. Introduction

Screening of phylogenetically diverse and unique organisms from rare or extreme ecosystems has been used to discover relevant bioactivities. In the 1960s, efforts to sample the deep ocean floor resulted in unexpected findings of high faunal diversity, turning the marine environment into a top spot for the search of biological active compounds. Up to now, bioactivities such as anti-tumor, anti-microtubule, anti-proliferative, photoprotective, as well as antibiotic and anti-fouling have been identified in the marine environment [1]. However, it was the disclosure of the role of bacteria in the production of bryostatins that opened the road for the biotechnological synthesis of anti-tumor compounds from marine origin [2,3] and triggered the interest in marine microbial resources from many biotechnological angles [4].

Nevertheless, to maximize the chemical diversity available from microorganisms, and their translation into suitable commercial products, new extreme sources of microbes are needed. Deep sea hydrothermal vents, with physical extremes of temperature (4 to 400 °C) and pressure, a complete absence of light and abrupt chemical, pH and temperature gradients, are considered one of the most extreme and dynamic environments on Earth. The most studied hydrothermal systems are located in the eastern Pacific and in the north-central Atlantic [5]. The Mid-Atlantic Ridge (MAR), that extends from the Arctic Ocean to the African Continent, is mainly constituted by submerged mountains [6] and harbours several hydrothermal fields such as Menez Gwen, Menez Hom, Rainbow, Lucky Strike and Mount Saldanha [7]. 

Despite the high scientific and commercial interest in the microbial ecology of these ecosystems, relatively little is known about the diversity of functional taxonomic groups of free-living microbes that occupy these niches as well as their biotechnological potential. Culture-dependent and culture-independent methods have been employed with the *Proteobacteria* phylum comprising approximately one-third of all known bacteria present at the deep sea hydrothermal vents [7,8,9].

**Figure 1:**
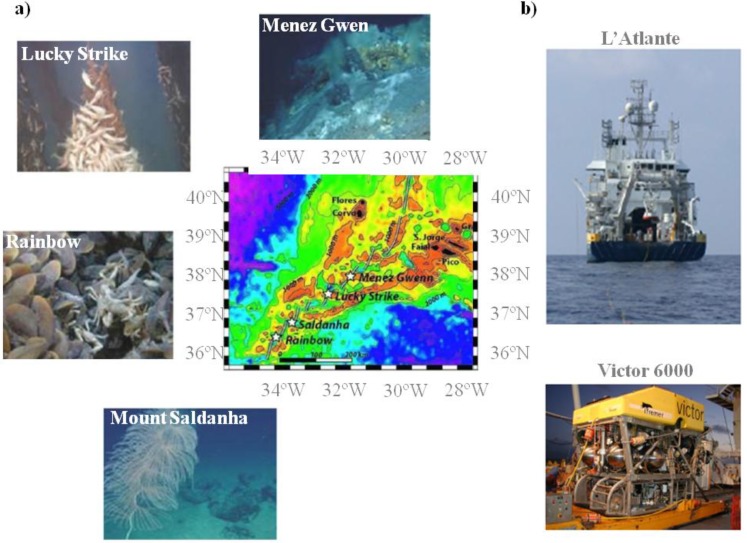
Portuguese mission SEAHMA-1 in MAR (Mid-Atlantic Ridge). (**a**) Localization and images of five hydrothermal vents visited during the missions: Rainbow, Mount Saldanha, Lucky Strike and Menez Gwen; (**b**) Images of the ship L’Atlante and of the ROV (Remote operating vehicle) Victor 6000 used in the expedition.

In this work, a marine prokaryotic collection from four MAR hydrothermal vents, Menez Gwen, Rainbow, Lucky Strike and Mount Saldanha (Figure 1) was created. Upon isolation of the marine prokaryotes, a polyphasic approach [10,11] was applied which allowed the definition of clusters of isolates and the identification of some prokaryotic species. In order to exploit the bioactivity potential of this unique collection, an industrially relevant sub-set of crude bacterial extracts was prepared and a yeast-based platform that relies on yeast susceptibility to UV radiation [12] was developed allowing the identification of deep sea crude extracts with photoprotection activity. It is known that although some UV radiation can have beneficial effects, as it stimulates vitamin D production [13], too much exposure to UV radiation is generally harmful and needs blockage. Examples of injuries caused by UV radiation include collagen fibre damage that accelerates skin aging and, ultimately, skin cancer [14]. Some compounds were already described acting as sunscreens, with the best known being the melanins present in humans and other animals and even in some bacteria, black yeasts and pigmented filamentous fungi [15,16,17]. In addition to melanins, other compounds such as scytonemins from cyanobacteria, mycosporines from fungi and mycosporine-like amino acids from cyanobacteria, algae and animals have been used to counteract photodamage [18]. Nevertheless, the discovery of new-generation natural sunscreens with improved characteristics such as a broad and intense absorption bands together with a high degree of photostability is of high interest and may be found in deep sea crude extracts.

## 2. Results and Discussion

### 2.1. New Marine Prokaryotes Isolates from MAR Vents

From the 36 samples collected from the MAR hydrothermal vents visited (Supplemental Table S1), two of them, MH1 and RB1 were used only for the isolation of yeasts that were described elsewhere [19], whereas all other 34 samples were used in this work for prokaryotes isolation. From the 34 samples initially inoculated, 25 collected from four MAR hydrothermal vents, allowed the isolation, and further growth in standardized culturing conditions, of 289 marine prokaryotes. Based on oxygen demand and growth temperature, three phenotypic operational groups were defined. Group I contains 140 psychrotolerant aerobes, group II contains 125 psychrotolerant anaerobes and group III contains 24 thermophilic anaerobes (Table 1). In relation to the standardized culturing conditions, microorganisms belonging to group I could be grown on Nutrient Broth supplemented with 3% sea salts at 22 °C for 72 h, group II microorganisms could be grown on the same media as group I but supplemented with 0.05% cysteine and incubated at 22 °C for 96 h whereas group III microorganisms could be grown on the same medium as group II although different incubation temperatures were needed for optimal growth.

**Table 1:** Distribution of the MAR vents isolates in each phenotypic operational group according to the type of sample (**a**) and hydrothermal vent (**b**) from which they were collected.

		Type of sample				
		Water	Animals	Sediments	Chimneys	Total
**Phenptypic operational group**	**I**	18	87	24	11	**140**
	**II**	11	56	44	14	**125**
	**III**	0	3	0	21	**24**
	**Total**	**29**	**146**	**68**	**46**	**289**
(**a**)						
		**Hydrothermal vent**				
		**Menez Gwen**	**Lucky Strike**	**Mount Saldanha**	**Rainbow**	**Total**
**Phenptypic operational group**	**I**	57	32	3	48	**140**
	**II**	82	15	0	28	**125**
	**III**	1	11	0	12	**24**
	**Total**	**140**	**58**	**3**	**88**	**289**
(**b**)						

### 2.2. MAR Vents Prokaryotic Biodiversity

**Figure 2:**
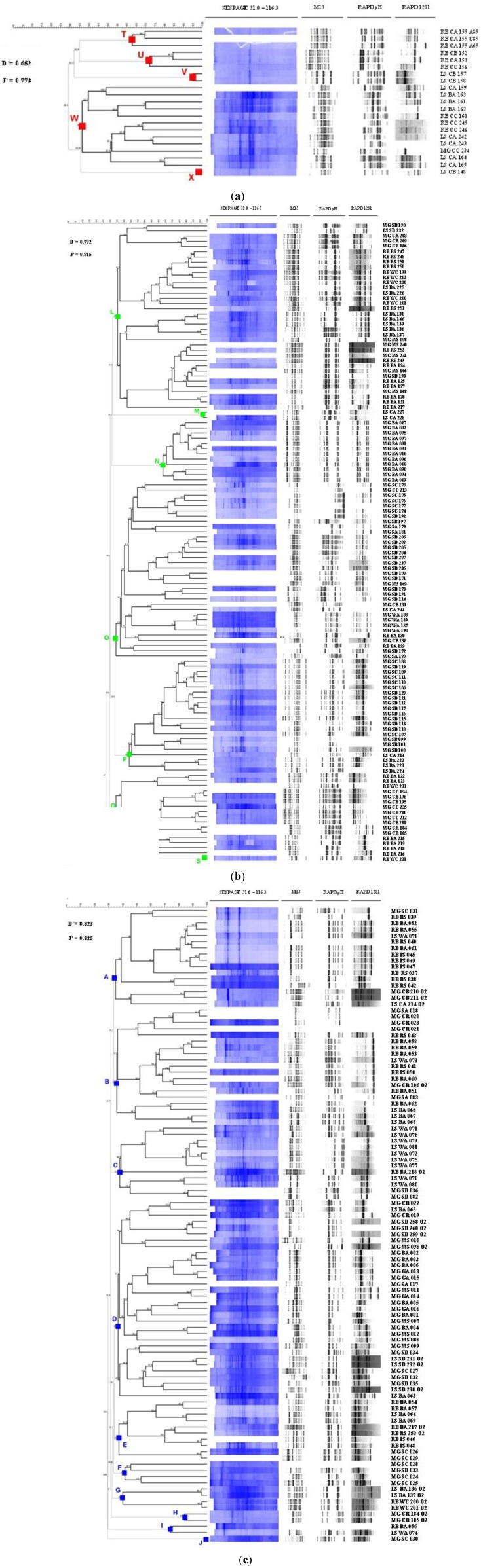
Consensus polyphasic dendrograms from the in silico analysis of the PCR fingerprinting profiles (primers csM13, pH and 1281) and whole-cell protein profiles obtained for Group III (**a**); Group II (**b**) and Group I (**c**). Cophenetic correlation coefficient, ρ=0.91.

246 isolates were further studied by allying a polyphasic characterization approach to a strain clustering strategy. The chemotaxonomic and molecular typing methods whole-cell protein profiling by SDS-PAGE and PCR fingerprinting were applied single and in combination. In relation to the PCR fingerprinting technique, minisatellite primer csM13 and random primers pH and 1281, targeting different genomic regions, were used. Using this last technique, 245 profiles were obtained with csM13-PCR fingerprinting, 244 profiles were obtained with pH-PCR fingerprinting and 233 profiles were obtained with primer 1281. The reproducibility of the fingerprinting profiles, expressed by the percentage of similarity obtained with 10% randomly chosen replicas, was 75% ± 12.4% for the M13 primer, 83% ± 10.5% for the pH primer and 81% ± 11.3% for the 1281 primer. From a composite dendrogram of PCR fingerprints, Shannon (J’) and Simpson (D’) biodiversity indexes of 0.836, 0.818 and 0.832 for J’ and 0.785, 0.805 and 0.643 for D’ were obtained for the operational groups I, II and III, respectively. Regarding the whole-cell protein profiling, the 246 isolates were firstly grown under standardized culturing conditions defined for each operational group, allowing the detection of 212 whole-cell protein profiles. The average reproducibility of the whole cell protein profiles, estimated as above, was of 81% ± 11.5%. Biodiversity indexes of 0.800, 0.561 and 0.596 for J’ and 0.643, 0.561 and 0.450 for D’ were obtained for the operational groups I, II and III, respectively. The combination of the two techniques allowed the construction of consensus polyphasic dendrograms for each phenotypic operational group with the subsequent identification of 23 related clusters of phenetically similar isolates (Figure 2). This multiple integrated approach has led to a more robust dataset showing that incorporation of data from multiple methods leads to consensus dendrograms with a better fit than the one obtained when each method is used alone. 10 clusters were identified in group I, 8 clusters in group II and 5 clusters in group III. The distribution of the isolates in each cluster, based on sampling site and type of sample, is presented in Table 2. Global biodiversity indexes of 0.826, 0.816 and 0.773 for J’ and 0.823, 0.702 and 0.652 for D’ were obtained for the operational groups I, II and III, respectively. Based on these indexes, it is also observed that the phenetic diversity decreases from group I to group III, which is in accordance with the number of clusters identified in each group. In relation to group III, it is likely that the low indexes of diversity obtained are due to the fact that this group contains the most difficult microorganisms to cultivate, which might act as a biodiversity bias. A sub-set of isolates from the recognized clusters were identified by 16S rRNA gene sequencing and phylogenetically allocated. Although the majority of the sequences analyzed belong to already known prokaryotic species, for 43% of the sequences only genus level homology was observed (data not shown). In these cases, the similarities values obtained with other known species were lower than 98%, indicative of a high probability that almost half of this collection is constituted by new prokaryotic species [20] making it very appealing for the search of new industrially relevant bioactivities.

### 2.3. A MAR Vents Bacteria Collection Suitable for Industry

In order to economically explore the biotechnological potential of the identified isolates, a sub-set of the collection was selected for commercial exploitation and technology transfer from academic research institutes (MAR vents biotech collection). The selected bacteria isolates were the ones whose industrial scale production would be economically viable, such as low salt concentrations supported (less than 4%), regular growth temperature (20–40 °C) and effective growth in the presence of oxygen. Optimized general conditions for laboratory growth, where highest yields are obtained in the shortest incubation time, were achieved for 139 psychrotolerant aerobic or facultative anaerobic isolates representative of all MAR vents (Table 3). In addition, stress growth conditions were also tested to further increase the diversity of secondary metabolites being produced. Furthermore, optimization of downstream extraction methods was also performed, allowing to obtain high amounts of dry weight aqueous extract per liter of culture in a sustainable manner. Currently, the MAR vents bacteria extract library comprises 484 extracts, including 212 organic extracts and 272 aqueous extracts. 

**Table 2:** Distribution of the isolates of each one of the 23 identified clusters according to the type of sample (**a**) and hydrothermal vent (**b**) from which they were collected.

		Type of sample			
Group	Cluster	Water	Animals	Sediments	Chimneys
**I**	**A**	1	11	1	3
	**B**	1	16	2	
	**C**	9	1	2	
	**D**		21	11	
	**E**		8	2	
	**F**			4	
	**G**	2	2		
	**H**		2		
	**I**	1	1		
	**J**			1	
**II**	**L**	5	28	3	
	**M**				2
	**N**		12		
	**O**	4	3	23	4
	**P**		3	18	1
	**Q**		5		7
	**R**		4		
**III**	**S**		1		
	**T**				3
	**U**				3
	**V**				2
	**W**		3		9
	**X**				1
(**a**)					
		**Hydrothermal vent**			
**Group**	**Cluster**	**Menez Gwen**	**Lucky Strike**	**Mount Saldanha**	**Rainbow**
**I**	**A**	3	2	11	
	**B**	6	4	9	
	**C**	2	9	1	
	**D**	24	5		3
	**E**	2	2	6	
	**F**	4			
	**G**		2	2	
	**H**	2			
	**I**		1	1	
	**J**	1			
**II**	**L**	10	8	18	
	**M**		2		
	**N**	12			
	**O**	31	1	2	
	**P**	18	4		
	**Q**	9		3	
	**R**			4	
**III**	**S**			1	
	**T**			3	
	**U**			3	
	**V**			2	
	**W**	1	8	3	
	**X**		1		
(**b**)					

**Table 3:** MAR vents biotech collection. MAR vents collection is composed of marine bacteria isolated from different types of samples and from the different hydrothermal fields.

Marine Strain	Vent	Type of sample	Depth (m)	T (°C)
MG BA 001 to 006	Menez Gwen	*Bathymordiolus azoricus*	825	8.4
MG MS 007 to 012		*Microcaris* sp.	825	8.2
MG GA 013 to 016		Gastropode	825	8.4
MG SA 017 and 018		Sediment A	825	8.7
MG CR 019 to 023		Crab	825	8.2
MG SC 024 to 036		Sediment C	869	8.3
RB RS 037 to 043	Rainbow	*Rimicaris* sp.	2296	3.7
RB PS 045 to 050		*Pachicara* sp.	2297	3.7
RB BA 051 to 062		*Bathymordiolus azoricus*	2290	3.8
LS BA 063 to 069	Lucky Strike	*Bathymordiolus azoricus*	1693	4.4
LS WA 070 to 081		Water	1728	4.3
MG SD 082	Menez Gwen	Sediment D	808	9.1
MG SA 083		Sediment A	825	8.7
MG MS 098O_2_		*Microcaris* sp.	825	8.2
RB BA 124O_2_, 125O_2_, 127O_2_, 128O_2_ and 131O_2_	Rainbow	*Bathymordiolus azoricus*	2290	3.8
LS BA 136O_2_, 137O_2_, 138O_2_, 139O_2_ and 146O_2_	Lucky Strike	*Bathymordiolus azoricus*	1693	4.4
MG MS 168O_2_ and 184O_2_	Menez Gwen	*Microcaris* sp.	825	8.2
MG CR 184O_2_, 185O_2_ and 186O_2_		Crab	825	8.2
MG CC 194O_2_		Chimney C	808	266.2
MG CB 195O_2_ and 196O_2_		Chimney B	865	266.2
RB WC 199O_2_, 200O_2_, 201O_2_ and 202O_2_	Rainbow	Water	2301	3.7
MG CR 203O_2_ and 209O_2_	Menez Gwen	Crab	825	8.2
MG CB 210O_2_ and 211O_2_		Chimney B	865	266.2
MG CC 212O_2_ and 213O_2_		Chimney C	808	266.2
LS CA 214O_2_	Lucky Strike	Chimney A	1728	231.3
RB BA 215O_2_, 216O_2_ , 217O_2_, 218O_2_ and 219O_2_	Rainbow	*Bathymordiolus azoricus*	2290	3.8
RB WC 220O_2_ and 221O_2_		Water	2301	3.7
LS BA 225O_2_ and 226O_2_	Lucky Strike	*Bathymordiolus azoricus*	1693	4.4
LS CA 227O_2 _and 228O_2_		Chimney A	1728	231.3
LS SD 230O_2_, 231O_2_ and 232O_2_		Sediment D	1691	4.3
MG CC 235O_2_	Menez Gwen	Chimney C	808	266.2
MG MS 240O_2_ and 241O_2_		*Microcaris* sp.	825	8.2
RB RS 247O_2_, 248O_2_, 249O_2_, 250O_2_,251O_2_, 252O_2_ and 253O_2_	Rainbow	*Rimicaris* sp.	2296	3.7
MG SD 258O_2_, 259O_2_ and 260O_2_	Mount Saldanha	Sediment D	2200	3.8

### 2.4. MAR Vents Bacteria Extracts Possess Photoprotection Capacity

**Figure 3:**
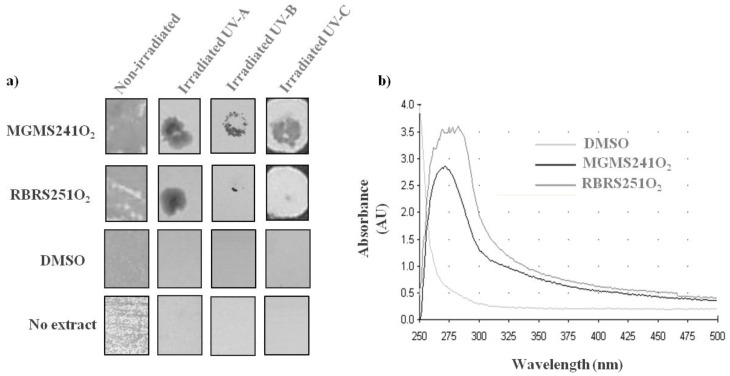
Anti-UV platform and extracts screening. (**a**) Results for yeast cell growth obtained after 3 days incubation at 30 °C of 3 plate sets: following non-irradiation and following either UV-A (365 nm), UV-B (312 nm) or UV-C (255 nm) lethal dose irradiation without or with the addition of a 2 to 4 µL drop of natural extracts from MGMS241O2 and RBRS251O2 isolates and also of a DMSO control; (**b**) UV-VIS spectra of natural extracts from MGMS241O2 and RBRS251O2 isolates and also of a DMSO control.

The development of anti-UV products showing high degree of photostability, capacity to be retained by the skin and the lack of allergenic potential is of high interest for several industries [17]. Searching in nature for products with such properties is an attractive strategy as a high number of microorganisms have already developed photoprotection and there are not yet available natural anti-UV protectors on the market. To explore MAR vents bacteria extracts photoprotection capacity, a yeast-based assay was developed subjecting the cells to either UV-A or UV-C minimum lethal dose of radiation in the presence or in the absence of the extracts. An extract with UV-protective capacity would allow the yeast cells to proliferate and form colonies, whereas a non protective extract would not allow cell growth. Firstly, a minimum lethal dose of 1.2 J/cm^2^ was identified for the UV-C lamp whereas a minimum lethal dose of 42 J/cm^2^ was the one identified for the UV-A lamp, being these the doses used in the screening. As a result, 2 out of the 24 tested aqueous extracts allowed yeast growth following a lethal dose of UV-A. In the case of UV-C, 16 out of the 57 tested aqueous extracts showed a radiation protection. Interestingly, 2 aqueous extracts, from MGMS241O_2_ and RBRS251O_2_ isolates, showed both anti-UV-A, and anti-UV-C protective capacity (Figure 3a). UV-B photoprotection of these two extracts was also observed, under a minimum lethal dose of 0.084J/cm^2^ (Figure 3a). In order to confirm that these extracts can be considered UV absorbers, absorption spectra were obtained with both extracts showing broad absorption spectra from 250 to at least 375 nm with a peak around 280 nm (Figure 3b).

**Figure 4:**
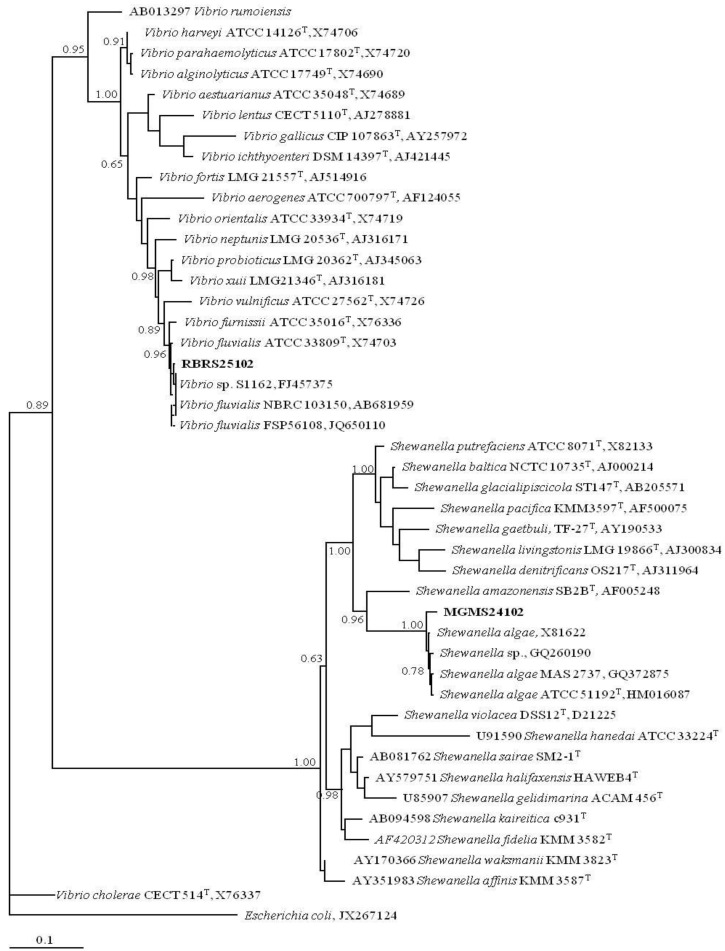
Phylogenetic tree (Bayesian MCMC method) obtained with 16S rRNA sequences (1250 nucleotide positions), corresponding to the sequences determined in this work, the most closely related ones retrieved from BLAST search and representatives of *Vibrio* and *Shewanella* genera. *Escherichia coli* was included to root the tree. Numbers associated to each node refer to probability values. Access numbers of GenBank sequences are indicated and names in bold face correspond to sequences determined in this work. T: type strain.

16S rRNA sequencing was then performed with MGMS241O_2_ being identified as a new strain belonging to the species *Shewanella algae* and RBRS251O_2 _as a new strain belonging to the species *Vibrio fluvialis* (Figure 4). Interestingly, as a nonfermenting facultative anaerobe, *Shewanella algae* has already been described as producing melanin to be used as an electron acceptor when oxygen concentration is low [21] but its role in photoprotection is much less studied. In the case of *Vibrio fluvialis*, to our knowledge, no photoprotection studied has been reported so far. In fact, melanin has been described as a virulence factor in pathogenic free-living strains of *Vibrio cholera* [22] and there are already some strains described as UV resistant [23] but no photoprotective activity neither photoprotective compounds have been described for *Vibrio fluvialis*. Hence, this is the first time that a photoprotection activity is identified for *Vibrio fluvialis* and that may be specific for this new isolated strain.

## 3. Experimental Section

### 3.1. Marine Bacteria Isolation and Culturing

During the Portuguese research mission SEAHMA-I, five MAR sites along the Azores archipelago (Menez Gwen, Lucky Strike, Mount Saldanha, Rainbow and Menez Hom) were monitored. Deep-sea sampling was performed using the submersible VICTOR 6000 and 36 samples were collected (Supplemental Table S1). Sample processing started on board using the experimental procedures depicted in Supplemental Figure S1 and several sea salts based culture media suitable for the isolation of marine aerobic and anaerobic bacteria (see Supplemental Table S2 for media composition) were used. After isolation, isolates were grown in a commercial culturing media (0.5% peptone (w/v), 0.3% meat extract (w/v)) supplemented with 3% sea salts. 

### 3.2. PCR Fingerprinting and Whole-Cell Protein Profiling and Phylogenetic Analysis

DNA was extracted from the isolates obtained in culture as described in [24] and stored at 4 °C. PCR fingerprinting with the minisatellite primer *cs*M13 (GAGGGTGGCGGTTCT) and random primers pH (AAGGAGGTGATCCAGCCGCA) and 1281 (AACGCGCAAC) were applied as described elsewhere [25]. Whole-cell protein (WCP) extracts were prepared according to [11] using cultures grown from 72 to 96 h in Nutrient Broth supplemented with 3% sea salts. WCP profiles were obtained by SDS-PAGE using a 10% acrylamide gel in a Tris-HCl buffer, 80 µg of total protein per lane and Coomassie blue staining for protein visualization. PCR fingerprints and WCP profiles were analyzed by hierarchical clustering with BioNumerics software (Applied Maths), using Pearson’s correlation coefficient and UPGMA agglomeration. Shannon evenness (H’) and Simpson diversity (D’) indexes [26,27] were used to assess biodiversity levels. Reproducibility of PCR fingerprinting and WCP profiling was estimated as the average percentage of similarity amongst clustered duplicates, using 10% of randomly chosen duplicates. Molecular identification was performed for a sub-set of isolates belonging to selected clusters. 16S rRNA gene was amplified with universal primers 21F and 1392R and DNA sequencing was performed using the same primers on the automated sequencer CEQ 2000-XL (Beckman Coulter, EUA). For phylogenetic analysis, sequence alignments were made with Clustal X [28] and visually corrected. The Bayesian Markov chain Monte Carlo (MCMC) method of phylogenetic inference [29] was applied to estimate phylogenetic relationships using MrBayes software [30].

### 3.3. Bacteria Collection Selection and Extracts Preparation and Analysis

A sub-set of the isolates was selected for commercial purposes. Psychrotolerant aerobic or facultative anaerobic bacteria from the original collection were chosen as representatives of the diversity of the collection. The selected isolates were adapted to controlled laboratory growth conditions and both aqueous and organic extracts were produced following standard protocols already described for microorganisms [31]. In particular for the case of aqueous extracts, 20 mL of pure water were added to each 3 to 5 g of wet biomass. Cells were then broken using a high pressure homogenizer and the produced aqueous extracts were lyophilized and re-suspended in DMSO at a concentration of 25 mg/mL.The UV absorbance measurement of the extracts was carried out using an ultraviolet-visible (UV-Vis) spectrophotometer (Evolution 300 Thermo Nicolet).

### 3.4. Anti-UV Yeast-Based Assay Development and Screening

Wild type BY4741 (*MATa: his3Δ0; leu2Δ0; met15Δ0; ura3Δ0*) yeast was plated on a single layer on YPD solid media. Yeast cells were pre-grown for 16 h at 30 °C in YPD liquid media with agitation and then diluted to an OD_600nm_ of 0.2. When exponential phase was reached, OD_600nm_ of 1, cells were ten-fold diluted and spread on an YPD solid media plate at a density of 2000 cells/cm^2^. For the UVC irradiation step a CAMAG UV lamp (255 nm) with an irradiance of 40 mW/cm^2^ was used at a distance of 5 cm from the plate. For the UVB irradiation step a UVITEC LF-115.M lamp (312 nm) with an irradiance of 1.2 mW/cm^2^ was used at a distance of 5 cm from the plate. For the UVA irradiation step a Hartmann UV-H lamp (365 nm) with an irradiance of 100 mW/cm^2^ was used at a distance of 2 cm from the plate. Minimal lethal dose of radiation of each of the lamps, which corresponds to the minimum dose needed for complete inhibition of yeast cell growth, was determined by increments in the radiation dose achieved through increases in the exposure time. For the screening, 2 to 4 µL of each aqueous extract at a concentration of 25 mg/mL in DMSO was added to the single-layer lawn of yeast cells and left to dry for a few minutes at room temperature. This was done in duplicate so that one set of plates was exposed to the minimal lethal dose of radiation, whereas the other set remained unexposed (control plates). Yeast plates were incubated at 30 °C up to 4 days and cellular growth was analyzed by counting the number of yeast colonies able to survive to the minimal lethal dose of radiation.

## 4. Conclusions

In recent years, numerous compounds from marine natural products were isolated possessing interesting biological activities, mainly for the pharmaceutical industry. At the moment, at least three compounds are in Phase III trials, seven compounds are in Phase II, three compounds are in Phase I and other numerous marine natural products are currently investigated for different applications [32]. However, these numbers are still below the expectations. In contrast to sponges, corals and other marine invertebrates, marine microorganisms, in theory, do not possess reproducibility and scale up limitations, being able to supply sufficient amounts of the pure substances. In addition, the marine environment is so extreme that it may have given rise to the evolution of metabolic pathways with microorganisms producing new chemical entities [33]. In fact, from 1997 to 2008 around 660 new marine bacterial compounds were identified, being reasonable to expect that many more interesting bioactivities from these microorganisms are still to be found [34].

With the aim of developing a novel and industrially suited marine bacteria collection, 139 marine bacteria were selected from the 289 MAR hydrothermal vents isolates and 484 extracts were produced. This collection potential was previously tested with 65% showing bioactivity against CNS targets or some immune disorders targets, as well as interesting cosmetic potential or even enzymatic activities such as cellulase, chitinase, xylanase, pullulanase, lipase, pectinases and mannanase (data not shown). In this particular study, photoprotective capacity was demonstrated for 22% of the aqueous extracts tested, which is of extreme interest. Because crude aqueous extracts were tested for bioactivities, the chemical nature of the bioactive compounds is still unknown. In the case of strain MGMS241O_2_, identified as *Shewanella algae*, one can speculate that melanin is probably the bioactive compound but in the case of strain RBRS251O_2_, identified as *Vibrio fluvialis*, the photoprotective compound still needs to be identified. The association of secondary metabolites production with anti-UV photoprotection in microorganisms has already been established [16,17,18], but mainly when these microorganisms are isolated from exposed surfaces. Hence, its role in deep sea isolated microorganisms is very intriguing and might be explained by two, not excluded, hypotheses. One is that although MAR vents bacterial strains were isolated at depths where no UV light is detected, these were brought to the surface and adapted to grow in laboratory conditions where some UV light is now present. This may have triggered the production of photoprotective compounds that although not enough for a full protection may potentially intercept a fraction of the UV radiation conferring photoprotection in the yeast-based assay. The other possibility is that the metabolites responsible for the photoprotection are compounds that have a different role in their natural environment but that in favorable conditions may serve as photoprotectives. This would not be a novelty since several microbial sunscreens have alternative physiological roles [16,17,18] and should be exploited as novel chemical structures with interesting properties and modes of action may be identified. These novel compounds are of high interest for both pharmaceutical and cosmeceutical industries, for instance, where protective skin natural compounds are extremely needed. For the cosmeceutical industry, in which products are intended to enhance health and beauty of the skin, the bacterial extracts described in this work are of particular interest as they can be added directly in its crude state to already formulated products. In addition to these two industries, also astrobiological research is on high demand for new bioactives that protect against UV-C, such as bacterial melanin [17], which increases MAR vents bacteria extracts commercial value.

Interestingly, until just a few decades ago, it was widely accepted that microbes from extreme environments would require complex cultivation methods and would not likely produce metabolites other than those required for survival. It is clear from this work that, in fact, many of those microbes can be cultivated under standard laboratory conditions and are capable of novel and interesting natural product synthesis with market potential applications. As the global market for marine biotechnology products and processes is expected to reach $4.1 billion by 2015, with growth rates of around 12% in Europe [35], the exploitation of this collection for other industrially relevant bioactivities is indeed economically very appealing and is being pursued.

## Supplementary Files

Supplementary File 1 Supplemental Information (PDF, 186 KB)
