# Supplementary material for: Photoprotective Bioactivity Present in a Unique Marine Bacteria Collection from Portuguese Deep Sea Hydrothermal Vents
Source: Mar Drugs. 2013 May 10;11(5):1506–23. doi: 10.3390/md11051506 (PMC3707158; doi:10.3390/md11051506)
Supplement: Supplementary File 1 — Supplemental Information (PDF, 186 KB) [file marinedrugs-11-01506-s001.pdf]

Supplemental Information

Table S1. Characteristics of the samples collected at MAR vents. The samples were characterized based on the vent, type of sample, collecting equipment, depth and temperature from where they were collected.

Reference	Code	Vent	Type of sample	Collecting equipment	Depth (m)	T (℃)
MG1	MG WA	Menez Gwen	Water	Insulated box	825	8.4
MG2	MG BA		*Bathymordiolus azoricus*	Insulated box	825	8.4
MG3	MG MS		*Microcaris sp.*	Aspirator	825	8.2
MG4	MG GA		Gastropode	Insulated box	825	8.4
MG5	MG SA		Sediment A	Tweezers	825	8.7
MG6	MG CR		Crab	Tweezers	825	8.2
MG7	MG BI		*Bathymordiolus azoricus* (liquid inside)	Insulated box	825	8.4
MG8	MG SB		Sediment B	Core	993	7.5
MG9	MG SC		Sediment C	Core	869	8.3
MG10	MG SD		Sediment	Tweezers	808	9.1
MG11	MG CA		Chimney A	Tweezers	865	266.2 *
MG12	MG CB		Chimney B	Tweezers	865	266.2 *
MG13	MG CC		Chimney C	Tweezers	808	266.2 *
MH1	MH WA	Menez Hom	Water	Aspirator	1800	4.5
MS1	MS SA	Mount Saldanha	Sediment A	Tweezers	2244	3.8
MS2	MS SB		Sediment B	Core	2198	3.8
MS3	MS SC		Sediment C	Core	2187	3.8
MS4	MS SD		Sediment D	Core	2200	3.8
MS5	MS WA		Water	Aspirator	2116	3.8
MS6	MS SE		Sediment E	Insulated box	2198	3.9
MS7	MS SF		Sediment F	Insulated box	2198	3.9
MS8	MS WH		Water (active hole)	Insulated box	2198	3.8
LS1	LS SD	Lucky Strike	Sediment	Core	1691	4.3
LS2	LS CA		Chimney A (external)	Tweezers	1728	231.3 *
LS3	LS CB		Chimney B (internal)	Insulated box	1728	231.3 *
LS4	LS CC		Chimney C (internal)	Tweezers	1728	231.3 *
LS5	LS BA		*Bathymordiolus azoricus*	Tweezers	1693	4.4
LS6	LS WA		Water	Insulated box	1728	4.3
RB1	RB WA	Raimbow	Water	Aspirator	2295	3.7
RB2	RB CA		Chimney A	Insulated box	2301	333.5 *
RB3	RB CB		Chimney B	Tweezers	2301	333.5 *
RB4	RB CC		Chimney C	Tweezers	2257	333.5 *
RB5	RB RS		*Rimicaris sp.*	Aspirator	2296	3.7
RB6	RB WC		Water (close to chimney)	Aspirator	2301	3.7
RB7	RB PS		*Pachicara sp.*	Aspirator	2297	3.7
RB8	RB BA		*Bathymordiolus azoricus*	Tweezers	2290	3.8

* Maximum temperature measured by ROV at the hydrothermal field.

Figure S1. Prokaryotes isolation procedure. The isolation procedure includes the processing of the samples, the enrichment for 6 weeks and the isolation and conservation of the marine bacteria collection.

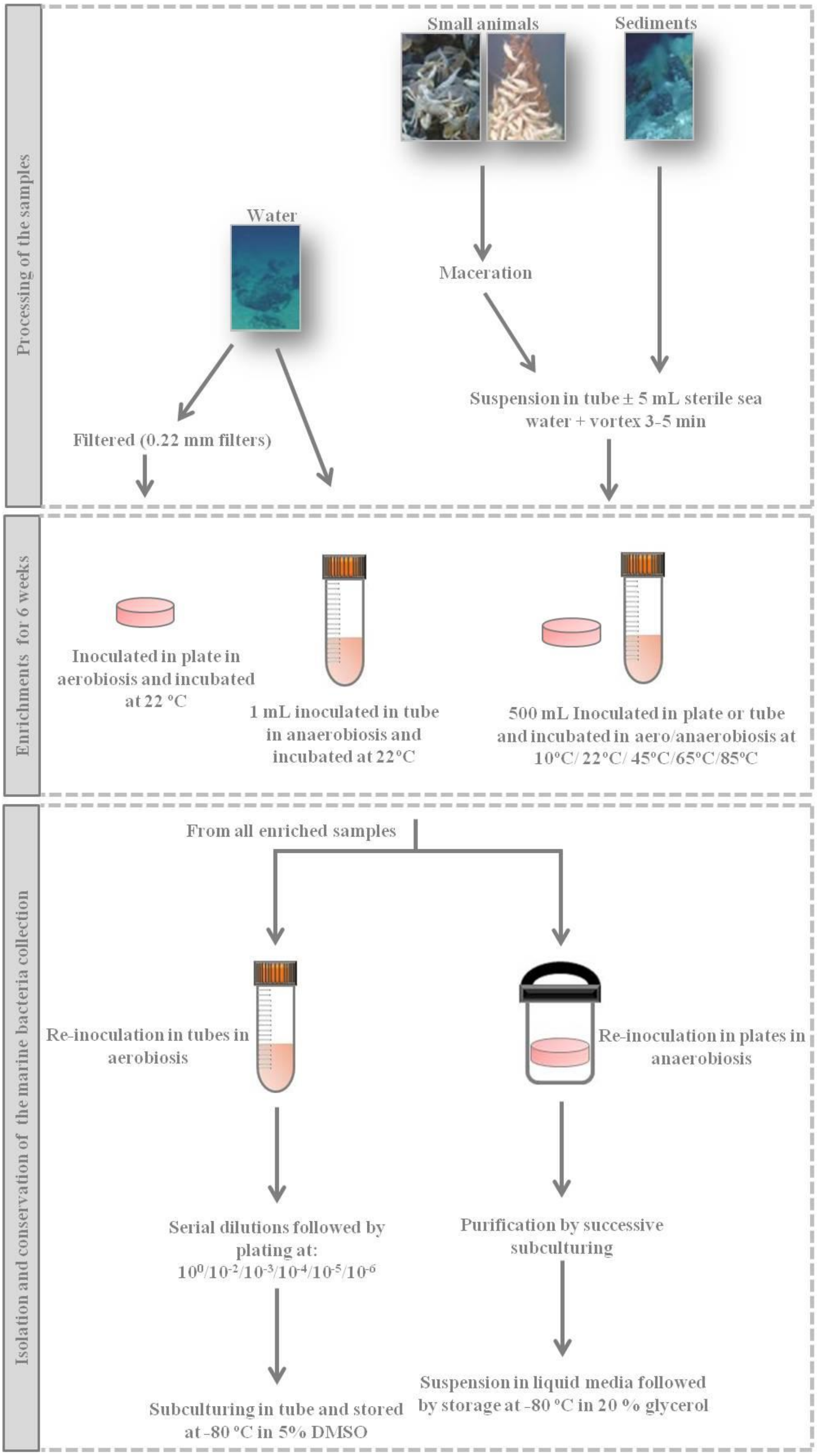

Table S2. Composition of media used for the isolation of marine bacteria and of the respective supplements. Two general media (M1 and M2) were used, together with a specific media for the isolation of sulphate-reducing bacteria.

**General Media M1**		**General Media M2**	
BHI	0.9% (w/v)	Peptone	0.1% (w/v)
PIPES	0.6% (w/v)	Cellobiose	0.5% (w/v)
Sea salts	3.0% (w/v)	Yeast extract	0.05% (w/v)
Sulfur	1.0% (w/v)	PIPES	0.6%
		Sea salts	3.0% (w/v)
		Sulfur	1.0% (w/v)

Both media were or not supplemented with sodium nitrate (0.17% (w/v)), iron sulphate (0.2% (w/v)) and manganese sulphate (0.085% (w/v)) in order to create M1S and M2S.

**Media for the isolation of sulphate-reducing bacteria**		**Trace elements solution**	
Yeast Extract	0.10%	Nitrilotriacetic acid	0.015%
Sodium Lactate	1.25%	$MgSO_4$	0.030%
Sodium acetate	0.14%	$MnSO_4$	0.005%
Sea Salts	3.00%	NaCl	0.010%
PIPES	0.60%	$FeSO_4$	0.001%
K_2HPO_4	0.05%	$CoCl_2$	0.001%
Na_2SO_4	0.40%	$CaCl_2$	0.001%
NH_4Cl	0.20%	$ZnSO_4$	0.001%
$MgSO_4$	0.20%	$CuSO_4$	0.100%
$CaCl_2$	0.02%	$AlK(SO_4)_2$	0.001%
$FeSO_4$	0.001%	H_3BO_3	0.001%
		Na_3MoO_4	0.001%
		$NiSO_4$	0.003%
		Na_2SeO_3	0.002%
		Na_2WO_4	0.002%

© 2013 by the authors; licensee MDPI, Basel, Switzerland. This article is an open access article distributed under the terms and conditions of the Creative Commons Attribution license (http://creativecommons.org/licenses/by/3.0/).
